# Evaluating diagnostic indicators of urogenital *Schistosoma haematobium* infection in young women: A cross sectional study in rural South Africa

**DOI:** 10.1371/journal.pone.0191459

**Published:** 2018-02-16

**Authors:** Hashini Nilushika Galappaththi-Arachchige, Sigve Holmen, Artemis Koukounari, Elisabeth Kleppa, Pavitra Pillay, Motshedisi Sebitloane, Patricia Ndhlovu, Lisette van Lieshout, Birgitte Jyding Vennervald, Svein Gunnar Gundersen, Myra Taylor, Eyrun Floerecke Kjetland

**Affiliations:** 1 Norwegian Centre for Imported and Tropical Diseases, Department of Infectious Diseases Ullevaal, Oslo University Hospital, Oslo Norway; 2 Institute of Clinical Medicine, University of Oslo, Oslo, Norway; 3 Department of Clinical Sciences, Liverpool School of Tropical Medicine, Liverpool, United Kingdom; 4 Department of Biomedical and Clinical Technology, Durban University of Technology, KwaZulu- Natal, South Africa; 5 Discipline of Obstetrics and Gynaecology, Nelson R Mandela School of Medicine, University of KwaZulu-Natal, Durban, South Africa; 6 Imperial College London, Claybrook Centre, London, United Kingdom; 7 Department of Parasitology, Leiden University Medical Centre, Leiden, Netherlands; 8 Section for Parasitology and Aquatic Diseases, Faculty of Health and Medical Sciences, University of Copenhagen, Copenhagen, Denmark; 9 Research Unit, Sorlandet Hospital, Kristiansand, Norway; 10 Department of Global Development and Planning, University of Agder, Kristiansand, Norway; 11 Discipline of Public Health Medicine, Nelson R Mandela School of Medicine, College of Health Sciences, University of KwaZulu-Natal, Durban, South Africa; University of the District of Columbia, George Washington University School of Medicine and Health Sciences, UNITED STATES

## Abstract

**Background:**

Urine microscopy is the standard diagnostic method for urogenital *S*. *haematobium* infection. However, this may lead to under-diagnosis of urogenital schistosomiasis, as the disease may present itself with genital symptoms in the absence of ova in the urine. Currently there is no single reliable and affordable diagnostic method to diagnose the full spectrum of urogenital *S*. *haematobium* infection. In this study we explore the classic indicators in the diagnosis of urogenital *S*. *haematobium* infection, with focus on young women.

**Methods:**

In a cross-sectional study of 1237 sexually active young women in rural South Africa, we assessed four diagnostic indicators of urogenital *S*. *haematobium* infection: microscopy of urine, polymerase chain reaction (PCR) of cervicovaginal lavage (CVL), urogenital symptoms, and sandy patches detected clinically in combination with computerised image analysis of photocolposcopic images. We estimated the accuracy of these diagnostic indicators through the following analyses: 1) cross tabulation (assumed empirical gold standard) of the tests against the combined findings of sandy patches and/or computerized image analysis and 2) a latent class model of the four indicators without assuming any gold standard.

**Results:**

The empirical approach showed that urine microscopy had a sensitivity of 34.7% and specificity of 75.2% while the latent class analysis approach (LCA) suggested a sensitivity of 81.0% and specificity of 85.6%. The empirical approach and LCA showed that *Schistosoma* PCR in CVL had low sensitivity (14.1% and 52.4%, respectively) and high specificity (93.0% and 98.0, respectively). Using LCA, the presence of sandy patches showed a sensitivity of 81.6 and specificity of 42.4%. The empirical approach and LCA showed that urogenital symptoms had a high sensitivity (89.4% and 100.0%, respectively), whereas specificity was low (10.6% and 12.3%, respectively).

**Conclusion:**

All the diagnostic indicators used in the study had limited accuracy. Using urine microscopy or *Schistosoma* PCR in CVL would only confirm a fraction of the sandy patches found by colposcopic examination.

## Introduction

Pathological changes caused by *Schistosoma haematobium* infection are found mainly in the urinary tract but are nearly as common in the gynaecological tract [1]. Urine microscopy is the most commonly used diagnostic method for *S*. *haematobium* [2]. However, this may lead to under-diagnosis of urogenital schistosomiasis in young women, as genital lesions may be present in the absence of ova in the urine [3, 4].

Females infected with *S*. *haematobium* may present with a range of urogenital symptoms, such as haematuria, dysuria, pelvic discomfort, vaginal discharge, vaginal bleeding, and post-coital bleeding [4, 5]. Furthermore, urogenital schistosomiasis has been hypothesised to be a co-factor in Africa’s HIV epidemic, where it has been estimated that individuals with schistosomiasis have 2–4 fold increased odds of having HIV infection [6, 7].

Urogenital morbidity in schistosomiasis is believed to be the result of the host’s local immune responses to *S*. *haematobium* ova in the urogenital tissues [8]. After the parasites enter the human host, the adult male and female worms couple and settle within veins surrounding the pelvic organs, including the urinary and genital systems [4, 9]. They lay ova that are either released into the environment by proteolytic migration through the urogenital mucosa or they get lodged in the urogenital organs, causing chronic inflammation [4, 10–12]. Pathological lesions known as sandy patches may appear in the urothelial mucosa of the bladder and in the epithelial mucosa of the vaginal wall and cervical surface [1, 13]. Lesions in the lower reproductive tract may be visualised by gynaecological investigation, for example using a colposcope [1, 13].

The prevalence and intensity of *S*. *haematobium* infection measured by urinary ovum excretion is age-dependent in endemic areas, with the highest prevalence and intensity occurring in children aged 10 to 14 years followed by a gradual decline to relatively low levels in adulthood [14].

Urine microscopy is relatively cheap, and easy to perform [2, 15]. However, it lacks sensitivity to detect light infections and is affected by day-to-day variation in ova excretion [2]. Biopsy of the sandy patches in the genital tract has been considered to be a good method for diagnosis of *S*. *haematobium* [16]. However, due to the co-endemicity of HIV and schistosomiasis, there is a concern that the iatrogenic lesion may cause an unnecessary increased risk of HIV transmission [4]. In a consensus meeting, experts in the field agreed that, in *S*. *haematobium* endemic areas, finding sandy patches in the genital tract, by colposcopy, is adequate for diagnosing female genital schistosomiasis in clinical practice [17]. However, even with advanced equipment, such as a colposcope, there are important limitations: colposcopy is an observer dependent diagnostic approach and the equipment is expensive [18], it may not be available in rural areas where the disease is prevalent and electricity is not available. Furthermore, lesions may be overlooked, for example in the fornices, or if ova are lodged too deep in the submucosa [19]. Holmen et al 2016 explored an objective method to diagnose genital lesions by using computer image analysis of the typical yellow colour seen in the sandy patches [20]. However, this method is still under development and has not yet been adapted for clinical use. The Polymerase chain reaction (PCR) method used in cervico-vaginal lavage (CVL) samples was found to be superior to detecting ova in Pap smears or wet mounts by microscopy [21–23]. However, the PCR method is currently not a realistic tool in rural clinics where *S*. *haematobium* is endemic, because it requires expensive laboratory equipment, steady funding and highly skilled personnel [2].

In the current study, we set out to explore the accuracy of four diagnostic indicators of urogenital *S*. *haematobium* infection in young women in an area endemic of *S*. *haematobium* in South Africa: (1) the urine microscopy for ova, (2) *Schistosoma* PCR of CVL, (3) the combined clinical and image analysis for sandy patches, and (4) the questionnaire on urogenital symptoms. First, we calculated the sensitivities and specificities of the available laboratory tests using a pseudo-gold standard. Then, we estimated these same quantities by fitting a latent class model to four diagnostic indicators without assuming a gold standard.

## Materials and methods

### Study area and recruitment

A cross-sectional study was carried out in rural high schools in three districts (Ilembe, uThungulu and Ugu) of KwaZulu-Natal, on the East Coast of South Africa, nested within a larger on-going study. The province is endemic for *S*. *haematobium* and we have previously reported that girls aged 10–12 years, from this same area, had a geometric mean intensity of 52 ova/10 ml of urine, indicating on-going transmission in the area [5].

Rural high schools in the study area were defined as the sampling frame. For practical reasons, high schools situated more than 2.5 hours drive from our study clinics in Otimati or Izotsha were excluded. This decision was made in order to ensure that participants could be fetched, investigated and returned home before dark, for safety reasons. The chosen clinic sites were almost in the middle of the districts and near the main road. There had to be enough space, water, electricity and security. For practical reasons, high schools with less than 300 pupils were excluded in order to save on the number of preparatory visits (minimum 4 visits per school, independent of potential participant number). The areas had to be endemic of schistosomiasis. Therefore, we only enlisted schools that were classified as rural by the Department of Education and were below the altitude of 400 meters above sea level, as indicated in an epidemiological study in the area by Appleton et al [24]. Furthermore, we only enlisted high schools with an estimated prevalence of *S*. *haematobium* of 10% or more, based on an initial show of hands for red urine in Ugu District and a haematuria dipstick survey in Ilembe and uThungulu districts [25].

**Fig 1:**
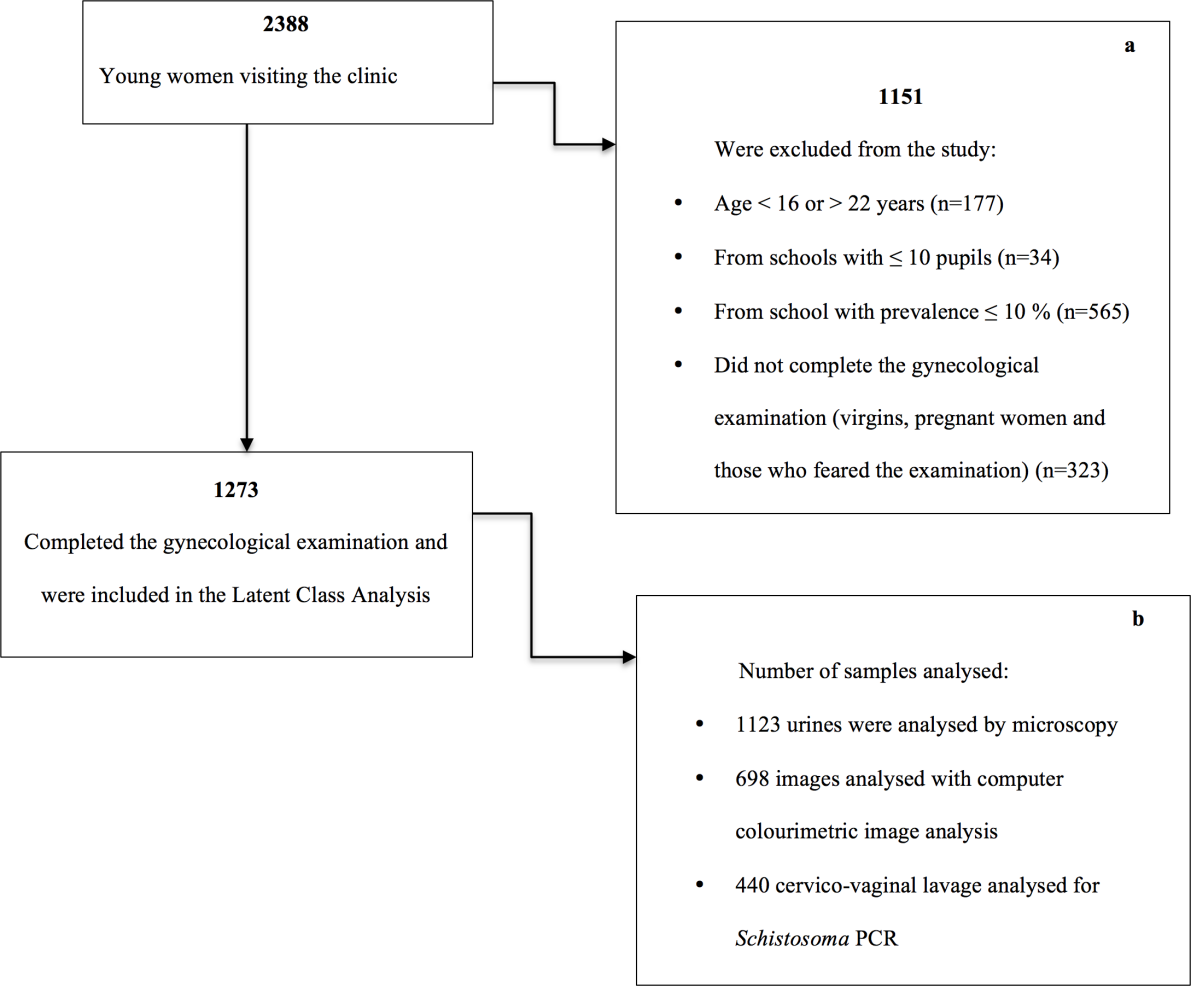
Flow chart showing the inclusion of participants. a. We were only able to calculate the school prevalence after all the pupils from a given school had been examined. Therefore we excluded a lot of the young women who visited the clinic. b. All were invited to provide samples. Only a selection of samples (34.6%) was sent for PCR analyses.

The recruitment took place from 2011 to 2013 (throughout the school year). Included schools were visited and general information was given on schistosomiasis. Dates for possible investigations were provided by the teachers and subsequently discussed with the young women individually. Parents were informed about the study. Sexually active young women aged 16–22 years who consented and underwent a full gynaecological examination were included. Pregnant women, virgins and non-consenting women were excluded (Fig 1).

### Questionnaire

A structured questionnaire was developed after reviewing the literature and was piloted among young school-going women (S1 File). The questionnaire was developed in English, translated into isiZulu (the local language) and then translated back into English to ensure accuracy of the translation. Questions were asked about current or previously experienced urogenital symptoms. For analysis purposes we combined any positive answer for nine urogenital symptoms associated with *S*. *haematobium* infection in young girls in KwaZulu-Natal province (i.e. abnormal discharge colour, abnormal discharge smell, burning sensation in the genitals, bloody discharge, genital ulcer, red urine, pain on urination, stress incontinence and urge incontinence) as a positive result, the negative cases reported none of these symptoms [5].

### Clinical examination

Prior to the gynaecological examination, the investigating clinician explained the procedure in detail to each participant and answered any questions related to the examination. The gynaecological examination started with visual inspection, followed by photocolposcopic examination using an Olympus OCS 500 colposcope with a mounted Olympus E420 (10 Mpx) single lens reflex (SLR) camera or a Leisegang colposcope with a mounted Canon EOS 650D (18 Mpx) SLR. Any observed lesions (e.g sandy patches) were documented on paper by the investigator, and later entered into electronic investigation forms by one data entry clerk, and if the cervix was visible, digital images were captured.

### Colourimetric computer image analysis

Images that fulfilled the inclusion criteria (cervix was in the field of view, there was no obstructive foreign material in the field of view and focus were adequate for visualization of the lesion) were analysed using computerised colourimetric image analysis to identify the characteristic sandy patches [20]. Briefly, the method identified areas of the cervix that were more yellow than the surrounding mucosa [20, 26]. The analysis is adaptive, allowing for a range of suboptimal imaging conditions such as over/under exposure and deviation in colour balance [20].

### Laboratory analysis

One urine sample was collected from each participant between 10 am and 2 pm as described previously [2]. Merthiolate-formalin solution (2%) was added to 10 mL of the sample. The sample was centrifuged and the pellet was deposited on two slides. Two independent technicians screened each of the slides for schistosoma ova by light microscopy [27]. CVL samples were collected by spraying 10 mL saline on the cervical surface 4 times, followed by drawing it back into the syringe [20]. CVL samples were stored at 4°C for a maximum of 4 days in the dark and thereafter stored at −80°C for several months. In a subsample, aliquots of 2 mL of CVL were transported to the Netherlands in frozen conditions for DNA isolation and Schistosoma DNA detection [22, 27, 28]. The real time PCR output consisted of a cycle threshold (Ct) value, indicating the number of cycles required before Schistosoma DNA was detectable [22]. A cut-off level for defining a positive diagnosis was defined by identifying the break-over point before the fluorescent signal plateaued [22]. The PCR analyses were performed blinded from microscopy results and other field data.

### Ethical considerations

All participants signed individual, written informed consent and their parents and guardians were informed about their participation in the study. The participants were made aware of their right to withdraw at any time during the study. The study was approved by the Biomedical Research Ethics Committee (BREC), University of KwaZulu-Natal (Ref BF029/07), KwaZulu-Natal Department of Health (Reference HRKM010-08) and the Regional Committee for Medical and Health Research Ethics (REC), South Eastern Norway (Ref 469-07066a1.2007.535). The Departments of Health and Education in Ugu, Ilembe and UThungulu districts, KwaZulu-Natal, gave permissions for this study. The ethical committees, BREC (annual renewal) and REC, were aware that minors (aged 16 and 17) were participating in the study and specifically approved independent minor consent without parental consent. As the study area is a schistosomiasis endemic area, all consenting participants were offered a single oral dose of 40 mg praziquantel/kg as recommended by the World Health Organization (WHO).

#### Statistical analyses

We began examining the diagnostic accuracy of (1) the combination of sandy patches and computerized image analysis as well as (2) urine microscopy, (3) *Schistosoma* PCR in CVL, and (4) self-reported urogenital schistosomiasis. We defined the empirical approach as the cross tabulation of the combined clinical observed sandy patches and computerized image analysis (as the pseudo-gold standard) and the three remaining diagnostic tests, calculating empirical sensitivities and specificities with 95% confidence intervals (CI) and negative and positive predictive values using Statistical Package for Social Sciences (SPSS) version 22 (IBM, Chicago, IL, USA).

To attempt to tackle the inherent problems with missing data and measurement error in the examined diagnostic tests, we employed a latent class analysis (LCA) model and full information maximum likelihood estimation. LCA allows grouping categorical data into classes via a probability model. Model based estimates of sensitivity and specificity for the tests of interest as well as a model estimate of prevalence can thus be provided through this approach.

In the LCA model, we assumed two latent classes: urogenital schistosomiasis negative and urogenital schistosomiasis positive, using all available data (including all four diagnostic tests with partially missing information). In order to attempt to ensure that the analyses would not be severely biased by the fairly large amount of included missing cases in the PCR analyses (only a few hundred samples could be sent overseas for analysis and of these, we had selected 50.0% of cases with clinically confirmed sandy patches), we initially sought to identify predictors of missingness. Through this process, we examined whether missingness would be fully accounted for by variables where there is complete information and if the partially missing information could be eventually incorporated in the LCA model. We finally evaluated the classification certainty of this model through entropy (where values of entropy near one indicate high certainty in classification and values near zero indicating low certainty).

LCA assumes that the relationships between the observed variables (i.e. diagnostic tests in the current study) are accounted for by their class membership and thus conditioning on class membership (i.e. the infection status in the current study) makes observed variables independent. We tested this assumption by speculating the standardized residuals for each response pattern from the four diagnostic tests as estimated from the LCA model. Further technical details of these models in the context of urinary schistosomiasis have been described elsewhere and thus they are not repeated here [29].

Statistical analyses were performed using either the Statistical Package for Social Sciences (SPSS) version 22 (IBM, Chicago, IL, USA) or the statistical software SAS version 9.3 (SAS Institute Inc., Cary, NC) while the LCA model was fitted using MPlus version 7.3 [30]. Venn diagrams were created using Venny version 2.1 (Juan Carlos Oliveros, http://bioinfogp.cnb.csic.es/tools/venny/).

## Results

### General characteristics of the study population

A total of 1237 young women aged 16–22 (median age 19) were included (Fig 1). One urine sample for microscopy was provided by 1123/1237 participants. Of these, 26.0% (292/1123) had detectable *S*. *haematobium* ova in the sample. In total 11.4% (50/440) had detectable *Schistosoma* DNA in CVL. Sandy patches were seen by the clinician in 21.9% (270/1233) of the participants and the computer image analyses detected sandy patches in 45.8% (319/696) of the participants, 62.2 (499/802) had sandy patches by either method. The rest were regarded as negative.

**Fig 2:**
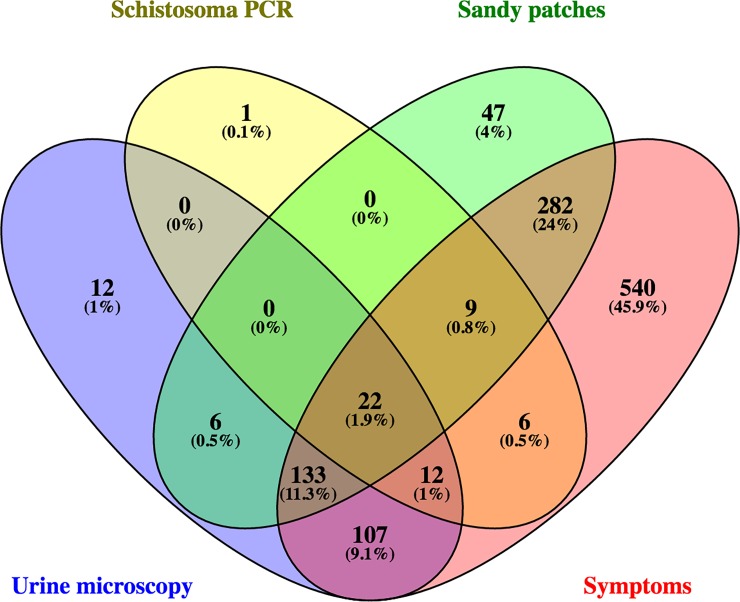
Venn diagram showing the overlap between positive findings in the four diagnostic indicators. The four diagnostic indicators were: (1) Urine microscopy, (2) Schistosoma PCR in cervico-vaginal lavagae, (3) sandy patches identified using clinical photocolposcopic examination or by computerised colourimetric image analysis and (4) self-reported urogenital symptoms: abnormal discharge colour, abnormal discharge smell, burning sensation in the genitals, bloody discharge, genital ulcer, red urine, pain on urination, stress incontinence and urge incontinence).

As many as 89.8% (1111/1237) had experienced one of the nine urogenital symptoms listed in Table 1. As shown in the table, abnormal discharge colour, pain on urination and urge incontinence were the most common urogenital symptoms. Fig 2 shows the overlap between the four diagnostic indicators (Fig 2).

**Table 1:** Urogenital symptoms reported by 1237 young women from an *S*. *haematobium* endemic area of rural South Africa.

Urogenital symptom ^a^	Prevalence (%)
Abnormal discharge colour	414/1233 (66.4)
Dysuria	689/1235 (55.8)
Urge incontinence	598/1235 (48.4)
Abnormal discharge smell	582/1231 (47.3)
Burning sensation in the genitals	554/1237 (44.8)
Red urine	424/1235 (34.3)
Stress incontinence	383/1234 (31.0)
Bloody discharge	289/1235 (23.4)
Genital ulcer	241/1234 (19.5)

a. Sorted by descending prevalence

Table 2 shows the characteristics of the young women who were and were not tested by *Schistosoma* PCR. Young women who were tested had significantly more sandy patches and urogenital symptoms than the untested young women.

**Table 2:** Comparing characteristics of women who were and were not tested by *Schistosoma* PCR.

	*Schistosoma* PCR tested ^a^	*Schistosoma* PCR not tested	P-value
Mean age	19	19	0.810 ^b^
*S*. *haematobium* by urine microscopy ^c^	24.3% (102/420)	27.0% (190/703)	0.311 ^d^
Sandy patch ^e^	75.6% (220/291)	54.6% (279/511)	< 0.001 ^d^
Exposed to unsafe water	95.2% (419/440)	95.9% (764/797)	0.602 ^d^
Urogenital symptoms ^f^	92.3% (406/440)	88.5% (705/797)	0.034 ^d^

Those who were to be tested by PCR (440/1237) were selected during recruitment. We sought to test sandy patch positive and negative in equal measure.

a. In-house *Schistosoma* polymerase chain reaction (PCR) [22].

b. Student’s T-test

c. *Schistosoma haematobium* ova detected in a single urine sample by microscopy [27].

d. Chi square test

e. Sandy patches identified using clinical photocolposcopic examination or by computerised colourimetric image analysis [17, 26]. The denominator is lower as not all who were tested by PCR had adequate images for computerised colourimetric image analysis.

f. Urogenital symptoms: abnormal discharge colour, abnormal discharge smell, burning sensation in the genitals, bloody discharge, genital ulcer, red urine, pain on urination, stress incontinence and urge incontinence [5].

### Diagnostic accuracy, assuming a pseudo-gold standard

Tables 3–5 show the sensitivity and specificity for urine microscopy, *Schistosoma* PCR of CVL and urogenital symptoms as indicated by the pseudo-gold standard. Having one or more of the urogenital symptoms was a sensitive indicator of sandy patches but showed low specificity, as expected. Similarly, using the four most common symptoms only (Table 1), the sensitivity and specificity were 81.6%, (95% CI: 77.9% - 84.9%) and 17.9% (95% CI: 13.7% - 22.7%), respectively. Using only the three most common symptoms (Table 1), the sensitivity was 79.4% (95% CI: 75.5% - 82.8%) and the specificity was 21.5% (95% CI: 17.0% - 26.6%).

**Table 3:** Cross tabulation of frequencies of negative and positive results for urine microscopy of *S*. *haematobium* compared to a pseudo-gold standard, with empirical sensitivities, specificities, positive and negative predictive values and 95% confidence intervals (CIs).

	Urine microscopy ^b^		
Pseudo gold standard ^a^	Negative	Positive	Total
**Negative**	203	67	**270**
**Positive**	303	161	**464**
**Total**	**506**	**228**	**734**
**Sensitivity (95% CI):** 34.7 (30.4 to 37.0) **Specificity (95% CI):** 75.2 (69.6 to 80.2)			

Missing data was not included in this analysis. CI: Confidence Interval

a. Sandy patches identified using clinical photocolposcopic examination or by computerised colourimetric image analysis [17, 26]

b. *Schistosoma haematobium* ova detected in a single urine sample by microscopy [27].

**Table 4:** Cross tabulation of frequencies of negative and positive results for *Schistosoma* PCR in cervico-vaginal lavage compared to a pseudo-gold standard, with empirical sensitivities, specificities, positive and negative predictive values and 95% confidence intervals (CIs).

	*Schistosoma* PCR in CVL ^b^		
Pseudo gold standard ^a^	Negative	Positive	Total
**Negative**	66	5	**71**
**Positive**	189	31	**220**
**Total**	**255**	**36**	**291**
**Sensitivity (95% CI):** 14.1 (9.8 to 19.4) **Specificity (95% CI):** 93.0 (84.3 to 97.7)			

Missing data was not included in this analysis. CI: Confidence Interval

a. Sandy patches identified using clinical photocolposcopic examination or by computerised colourimetric image analysis [17, 26]

b. In-house *Schistosoma* polymerase chain reaction (PCR) [22].

**Table 5:** Cross tabulation of frequencies of negative and positive results for self-reported urogenital symptoms compared to a pseudo-gold standard, with empirical sensitivities, specificities, positive and negative predictive values and 95% confidence intervals (CIs).

	Urogenital symptoms ^b^		
Pseudo gold standard ^a^	Negative	Positive	Total
**Negative**	20	209	**229**
**Positive**	25	329	**354**
**Total**	**45**	**538**	**583**
**Sensitivity (95% CI):** 89.4% (86.3% to 91.2%) **Specificity (95% CI):** 10.6% (7.3% to 14.6%)			

Missing data was not included in this analysis. CI: Confidence Interval

a. Sandy patches identified using clinical photocolposcopic examination or by computerised colourimetric image analysis [17, 26]

b. Any one or more of the following symptoms: abnormal discharge colour, abnormal discharge smell, burning sensation in the genitals, bloody discharge, genital ulcer, red urine, pain on urination, stress incontinence and urge incontinence [5].

In order to explore genital and urinary schistosomiasis together (“urogenital” schistosomiasis), urinary microscopy was entered into the pseudo-gold standard for amended analysis of Tables 4 and 5. We found that sensitivity and specificity remained unchanged (*Schistosoma* PCR in CVL: sensitivity 15.1%, (95% CI: 10.70% - 20.47%) and specificity 98.3%, (95% CI: 90.76% - 99.96%). Urogenital symptoms: sensitivity 89.1%, (95% CI: 86.11% - 91.60%) and specificity 9.4%, (95% CI: 5.73% - 14.23%).

### Diagnostic accuracy of *S*. *haematobium* infection indicators through an LCA model in the absence of a gold standard

We identified two predictors of missingness for the PCR analyses: sandy patches (p < 0.001) and urogenital symptoms (p = 0.035). The missing values of the PCR analysis were therefore fully accounted for by these two variables. Table 6 shows LCA model-based estimates of four diagnostic indicators for *S*. *haematobium* infection. In the LCA model, the estimated prevalence of urogenital schistosomiasis was found to be 17.3% (9.8–24.8%).

**Table 6:** Latent class analysis estimates of sensitivity and specificity with 95% confidence interval for four diagnostic tests (n = 1237).

Diagnostic indicator	Sensitivity % (95% CI)	Specificity % (95% CI)
Urine microscopy ^a^	81.0 (60.6 to 100.0)	85.6 (80.6 to 90.7)
*Schistosoma* PCR in CVL ^b^	52.4 (33.2 to 73.6)	98.0 (94.5 to 100.0)
Sandy patches ^c^	81.6 (70.3 to 92.9)	42.4 (37.9 to 47.0)
Urogenital symptoms ^d^	100 ^e^	12.3 (10.1 to 14.5)

CI: Confidence Interval.

a. *Schistosoma haematobium* ova detected in a single urine sample by microscopy.

b. In-house *Schistosoma* polymerase chain reaction (PCR).

c. Sandy patches identified using clinical photocolposcopic examination or by computerised colourimetric image analysis.

d. Self-reported urogenital symptoms: abnormal discharge colour, abnormal discharge smell, burning sensation in the genitals, bloody discharge, genital ulcer, red urine, pain on urination, stress incontinence and urge incontinence.

e. CI was not available as this probability was automatically fixed to a very high value during the estimation through the Mplus Software and thus no confidence intervals are estimated for a fixed value

### In a clinical setting

The research was performed in a clinical set ting in an *S*. *haematobium* endemic area and the above data suggest that urogenital symptoms would be a sensitive indicator of UGS. However, assuming the combined sandy patch diagnosis as the pseudo-gold standard, we explored how urine microscopy and *Schistosoma* PCR in CVL would perform in this clinical setting. For patients presenting with urogenital symptoms (high sensitivity), had we used urine microscopy as a confirmatory specific test, we would have diagnosed 37.4% (155/415) of the cases. If we had used *Schistosoma* PCR as a confirmatory specific test, we would have diagnosed 15.3% 31/203 of the cases.

## Discussion

In young women from an *S*. *haematobium* endemic area in rural South Africa, we compared four diagnostic indicators of urogenital schistosomiasis. We found that none of the four were satisfactory on their own. If a questionnaire on urogenital symptoms were used as a first sensitive screening tool and urinary microscopy for *S*. *haematobium* ova were used as a confirmatory test, at least two thirds of the young females in this study area with sandy patches in lower reproductive tract would have been missed. In our opinion, this would not be acceptable and if urine is negative a colposcopic examination must be done.

Each of the four different diagnostic indicators, (1) the urine microscopy for ova, (2) *Schistosoma* PCR of cervico-vaginal lavage, (3) the combined clinical and image analysis for sandy patches, and (4) the questionnaire on symptoms have limitations. The computer colour image analyses detected considerably more yellow areas than the clinicians, indicating that sandy patches may have been over- or under-diagnosed. The yellow colour of the mucosal surface could be caused by other diseases such as a late phase of herpes simplex [1]. Furthermore, for logistical reasons we only collected one urine sample per person. Due to the well known day-to-day variation in ova excretion, there must be a number of false negative cases in our study population resulting in an underestimation of the true prevalence [2]. Furthermore, post mortem studies indicate that ova are equally distributed in all genital organs and our sampling could not reach e.g. the Fallopian tubes [4, 31, 32]. Furthermore, PCR was performed in less than half of the cases, therefore sample size was low for the empirical analysis. Moreover, several studies indicate that sandy patches may constitute old, calcified ova without intact DNA, and this could render the *Schistosoma* PCR false negative even if sandy patches are present and even whilst symptoms remain [12, 23].

The large discrepancy in sensitivity estimates for urine microscopy in empirical and LCA approach is most possibly due to the fact that the two approaches are limited by different assumptions: The first assumes a gold standard (i.e. 100% sensitivity and specificity) but there are measurement errors and missing data; the LCA is based on the assumption of conditional independence of examined tests and includes partially missing data supposing that missingness would be fully accounted for by variables where there is complete information, a further limitation of the study. Estimated specificity was also better when evaluated by LCA than by empirical analysis. The entropy of the LCA model was estimated to be 0.612, which indicates that this might not the best model. More diagnostic tests might be needed to acquire more unbiased estimates of diagnostic accuracy through LCA. Standardized residuals for each response pattern from the four diagnostic tests from this model were not always between -2 and 2, which indicates that the assumption of local independence of the four diagnostic tests might not hold. Alternatively, better solutions might be acquired through more advanced latent variable models that relax the conditional independence assumption but this is beyond the principal aim of this study. Hence the results must be interpreted with caution.

As expected, both analytical approaches showed low specificity and high sensitivity of the questionnaire for urogenital symptoms. From a statistical point of view, although the sensitivity of this long list of symptoms was automatically estimated and fixed to 100%, we consequently have no confidence intervals associated with this estimate and thus no indication of how uncertain this result might be. To improve this result, other frameworks like for instance Bayesian inference for latent class models, might give improved solutions but such work is beyond the scope of the current research.

Schistosomiasis endemic populations represent a continuum between active infection with ova excretion (with or without symptoms and genital lesions) and women with calcified sandy patches who have no ovum excretion and some who may not even harbour live worms [8]. As shown in the Venn diagram (Fig 2), there is considerable overlap between the diagnostic indicators in the study population. From a diagnostic point of view, we can assume two populations within the cases: (1) those with ovum-laying worms and recent exposure and (2) those with urogenital consequences of previous ovum-laying [33]. The term “urogenital” covers both groups [34]. However, the diagnostic indicators will perform differently in the two groups; in group 1, ova counts and haematuria should be very good tools for diagnosis. However, in group 2, when the worms are dead and calcified ova pose a continuous “irritation” in the genital mucosa, visual inspection (colposcopy) and digital image analyses may be the only tools for diagnosis. In this study we could not differentiate between the two groups. An extensive array of tests would be needed to overcome this limitation, such as Circulating Anodic Antigen (CAA detects live worms), serology (detects current or previous infection), multiple urine and stool analyses, detection of lesions and biopsies. The latter represents an ethical problem [4].

In screening large numbers of patients, the diagnostic tools should be sensitive rather than specific, as this would minimize the risk of leaving infected patients without treatment [35]. A simple questionnaire may be relatively cheap, does not require any expertise, is less time-consuming and requires minimum training [36]. However, the listed symptoms may be caused by a long list of diseases and for clinical settings, and a confirmatory test is necessary lest we abuse scarce resources. Unfortunately, the current study shows that we will only be able to confirm a fraction of the urogenital schistosomiasis cases if we use urine microscopy or lavage PCR. This means that cases will likely be misdiagnosed and potentially devastating information will be given to the patient, such as “you likely have a sexually transmitted disease”. Further research is needed to explore if microscopy of several urines would increase sensitivity. Kjetland et al. in 2009, also found that *Schistosoma* PCR in lavage had a low sensitivity and high specificity [23]. The low yield of PCR in our study population indicates that other methods are needed.

### Conclusions

*S*. *haematobium* may cause morbidity in the urinary or genital tracts from the time of the infection, through the ovum-laying period and for decades after he immunological reaction to the dead and calcified ova [8]. In our population, even though they were all adolescents, they probably represented a continuum from active to past infections as the exposure to fresh water happened at different ages [36]. The different manifestations and durations of urogenital schistosomiasis could not be detected in a consistent fashion and all the diagnostic indicators used in the study therefore had limited accuracy. Further studies are needed to explore a sensible approach to the diagnosis of urogenital schistosomiasis. Furthermore, in order to develop cheap and reliable diagnostic tools, we need a better understanding of the underlying pathophysiological mechanisms that are responsible for the urogenital morbidity of *S*. *haematobium* infection.

## Supporting information

S1 File Questionnaire. The questionnaire included questions on current and previously experienced urogenital symptoms and water contact.
(DOCX)

S1 Table Dataset. The dataset used for the analyses.
(SAV)
