# Supplementary material for: Evaluating diagnostic indicators of urogenital Schistosoma haematobium infection in young women: A cross sectional study in rural South Africa
Source: PLoS One. 2018 Feb 16;13(2):e0191459. doi: 10.1371/journal.pone.0191459 (PMC5815575; doi:10.1371/journal.pone.0191459)
Supplement: S1 File — The questionnaire included questions on current and previously experienced urogenital symptoms and water contact. (DOCX) [file pone.0191459.s001.docx]

Supporting information S1 - Questionnaire

**URINE**

|  | **Wake waba nayo inkinga noma yiphi ngokuchama njenge:** / Have you ever had any problems with urination like: | **Esontweni eledlule /** This last week | **Kudala phambilini /** Sometime before | **Akukaze /** Never |
| --- | --- | --- | --- | --- |
| **a.** | **Zinhlungu uchama** / Pain when you urinate |  |  |  |
| **b** | **Ukuzwa sengathi ufuna ukuchama esikhaleni noma uhlezi unganyakazi uze ucishe uzichamele** / Sudden urge to urinate causing a leak, even when sitting still [urge incontinence] |  |  |  |
| **c.** | **Ukuzwa sengathi ufuna ukuchama esikhaleni noma unganyakazi ngaphandle kokuthi ucishe uzichamela** / Sudden urge to urinate, even when sitting still [urge] (no leakage) |  |  |  |
| **d.** | **Iconsi lomchamo uma ugxuma, ukhwehlela noma uhleka** / Drop of urine if you jump, cough or laugh [stress incontinence] |  |  |  |
| **d.** | **Umchamo obomvu** / Red urine |  |  |  |

# WATER CONTACT

1. **Manje ngizokubuza ngezinhlobo zezinto ozenzayo noma owake wazenza ngamanzi, uzenza noma wazenza kangakanani, uhlala isikhathi esingakanani emanzini nokuthi umzimba uthintana kangakanani namanzi.** /Now I will ask you what kind of water activity you have or have had, how often you do them, for how long you stay in the water and how much of your body that is in contact with the water:
   **Umfula**/river **Amadamu**/dam **Amanzi amile**/standing water. **Amanzi avela kulezizisuka**/ water from these sources. **None**
2. **Kukhona isikhathi empilweni yakho owake wahlangana nalamanzi?** [yebo] **[cha]**Is there a period in your life when you had water contact? (make crosses for each period)

   |___|___|___|___|___|___|___|___|___|___|___|___|___|___|___|___|___|___|___|___|___|___|
    1 2 3 4 5 6 7 8 9 10 11 12 13 14 15 16 17 18 19 20 21 22 yrs

# SYMPTOMS AND EVENTS

1. **Kubukeka kunjani ukuphuma ngaphansi kuwena? /** How is your current discharge?
   1. Kumbala muni/ Colour 1 2 3 4 5 6 7 8 [NA=99] (please circle)
   2. Okusagazana / Trace of blood 0 1 2 3 4 5 6 [NA=99]

| 1. **Wake wezwa ukungaphatheki kahle esithweni sakho sangasese njengo: /** Have you previously felt any discomfort in your private parts like**:** | | **Esontweni eledlule /** This last week | **Kudala phambilini** Sometime | **NA /** Never |
| --- | --- | --- | --- | --- |
| **a.** | **Ukuluma** / Itch |  |  |  |
| **b.** | **Ukushisa / Ukushoshozela** / Burn / Sting |  |  |  |
| **d.** | **Isilonda /** Sore / ulcer |  |  |  |
| **e.** | **Isimila / isigaxa** / Lump / tumour |  |  |  |
| **f.** | **Okuphumayo okusagazana /** Bloody discharge |  |  |  |
| **g.** | **Okushubile/ Okusasigaxana okuphumayo** Thick/ lumpy discharge |  |  |  |
| **h.** | **Okusamanzi okuphumayo /** Watery discharge |  |  |  |
| **i.** | **Iphunga elingajwayelekile /** Abnormal smell |  |  |  |
| **j.** | **Okuphumayo okunombala ongajwayelekile /** Abnormal coloured discharge |  |  |  |
| **k.** | **Kukhona osuke wakushutheka esithweni sakho sangasese njenge nsipho?.** / Did you put substances inside your vagina? Such as soap ? |  |  |  |
| **l.** | **Wake wagcatshwa esithweni sangasese noma waxilongwa ngezinsimbi ezicijile** / Ever had genital cutting or used a sharp instrument in the genital area (not episiotomy) |  |  |  |
| **m.** | **Ubuhlungu besinye** / Lower abdominal pain |  |  |  |
| **n.** | **Ubuhlungu ngaphansi kwezimbambo** / Upper abdominal pain |  |  |  |
